# Microstructure Evolution and Mechanical Properties Improvement in Liquid-Phase-Sintered Hydroxyapatite by Laser Sintering

**DOI:** 10.3390/ma8031162

**Published:** 2015-03-17

**Authors:** Songlin Duan, Pei Feng, Chengde Gao, Tao Xiao, Kun Yu, Cijun Shuai, Shuping Peng

**Affiliations:** 1 Hunan Provincial Tumor Hospital and the Affiliated Tumor Hospital of Xiangya School of Medicine, Central South University, Changsha 410013, China; 2 State Key Laboratory of High Performance Complex Manufacturing, Central South University, Changsha 410083, China; E-Mails: airyflier@csu.edu.cn (S.D.); fengpei@csu.edu.cn (P.F.); gaochengde@csu.edu.cn (C.G.); 3 Orthopedic Biomedical Materials Institute, Central South University, Changsha 410083, China; E-Mail: xiaotaoxyl@163.com; 4 Department of Orthopedics, the Second Xiangya Hospital, Central South University, Changsha 410011, China; 5 School of Materials Science and Engineering, Central South University, Changsha 410083, China; E-Mail: yukun2010@csu.edu.cn; 6 School of Basic Medical Science, Central South University, Changsha 410078, China

**Keywords:** liquid phase sintering, laser sintering, microstructure, mechanical properties, hydroxyapatite

## Abstract

CaO-Al_2_O_3_-SiO_2_ (CAS) as a liquid phase was introduced into hydroxyapatite (HAp) to prepare bone scaffolds. The effects of the CAS content (1, 2, 3, 4 and 5 wt%) on microstructure and mechanical properties of HAp ceramics were investigated. The optimal compression strength, fracture toughness and Vickers hardness reached 22.22 MPa, 1.68 MPa·m^1/2^ and 4.47 GPa when 3 wt% CAS was added, which were increased by 105%, 63% and 11% compared with those of HAp ceramics without CAS, respectively. The improvement of the mechanical properties was attributed to the improved densification, which was caused by the solid particle to rearrange during liquid phase sintering. Moreover, simulated body fluid (SBF) study indicated the HAp ceramics could maintain the mechanical properties and form a bone-like apatite layer when they were immersed in SBF. Cell culture was used to evaluate biocompatibility of the HAp ceramics. The results demonstrated MG-63 cells adhered and spread well.

## 1. Introduction

Bone grafts are frequently required to repair skeletal defects due to disease, trauma and congenital defects [1,2]. Bone scaffolds are one of the three key elements for bone grafts [3]. There is an urgent need in bone repair field to develop biological applicable scaffolds, which could guide bone regeneration in defects [4]. To fulfill bone repair requirements, scaffolds should possess bioactivity, biocompatibility and porous structure, and provide a structural support for the growth of cells and regeneration of tissues [5,6,7,8,9].

Hydroxyapatite [Ca_10_(PO_4_)_6_(OH)_2_, HAp] with solid [10,11] or hollow shape [12,13,14] is widely used calcium phosphate as a scaffold material in clinical application because of its similar mineral component to the human nature bone [15,16,17,18]. Several recent studies have been performed and show that HAp ceramics possesses excellent bioactivity and biocompatibility [10,11,12,19]. Besides, selective laser sintering (SLS) is an effective way to develop porous scaffolds with an intricate and controllable internal structure [4,20]. Moreover, it is hard to achieve a high densification by using SLS because of the short interaction time (0.2–200 ms) between the laser beam and material powders in the process of sintering. In general, the densification has direct effects on the mechanical properties of ceramics [21].

Liquid phase sintering (LPS) is an effective approach to improve the densification [22]. It involves three overlapping processes: (a) crystallite rearrangement; (b) coarsening by solution-reprecipitation; and (c) grain coalescence [23]. In the processes, a close spatial arrangement is obtained due to the formation of capillary force [24]. In recent years, LPS has been used to improve the mechanical properties of ceramics. Mikinori Hotta *et al.* [25] sintered SiC ceramics using AlN­Y_2_O_3_ as a liquid phase and found that flexural strength was increased to 1000 MPa by improving the densification. Defu Liu *et al.* [26] reported that the fracture toughness and compression strength of β-TCP scaffolds were increased by 18.18% and 4.45% using poly-L-lactic acid (PLLA) as a liquid phase, respectively, compared with those of β-TCP scaffolds without PLLA. Uma Batra *et al.* [27] reported that the sintered density and compression strength of HAp composites were improved using CaO-Na_2_O-P_2_O_5_-based additives as liquid phase.

Various oxides such as calcium oxide (CaO), alumina (Al_2_O_3_) or silicon oxide (SiO_2_) are the representative additives that have been widely used in HAp based ceramics [28,29]. The presence of small amounts of CaO, Al_2_O_3_ and SiO_2_, which form liquid phases in the grain boundaries, can affect sintering behavior and thus enhance the densification [30]. Moreover, silicon (Si) ion is beneficial to cells growth, mineralization of calcined tissues and bone calcification [31]. In this paper, the CaO-Al_2_O_3_-SiO_2_ (CAS) ternary-oxide system was selected as a liquid phase to sinter the HAp ceramics. The effects of CAS content on the microstructure and mechanical properties of HAp ceramics were investigated. The *in vitro* bioactivity and biocompatibility were evaluated using simulated body fluid (SBF) and MG-63 cells culture experiments, respectively.

## 2. Results and Discussion

### 2.1. Microstructural Analysis

**Figure 1:**
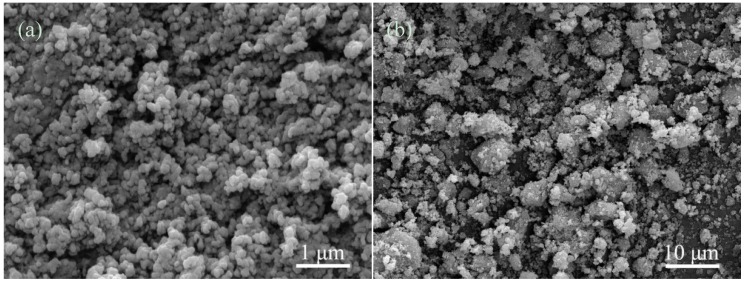
The initial powders: (**a**) hydroxyapatite (HAp); and (**b**) CaO-Al_2_O_3_-SiO_2_ (CAS).

The micromorphologies of the initial powders were displayed in Figure 1. The HAp powders showed a granule shape with an average size of 20 nm (Figure 1a). The CAS powders have a particle size range of 0.5–5 μm (Figure 1b).

**Figure 2:**
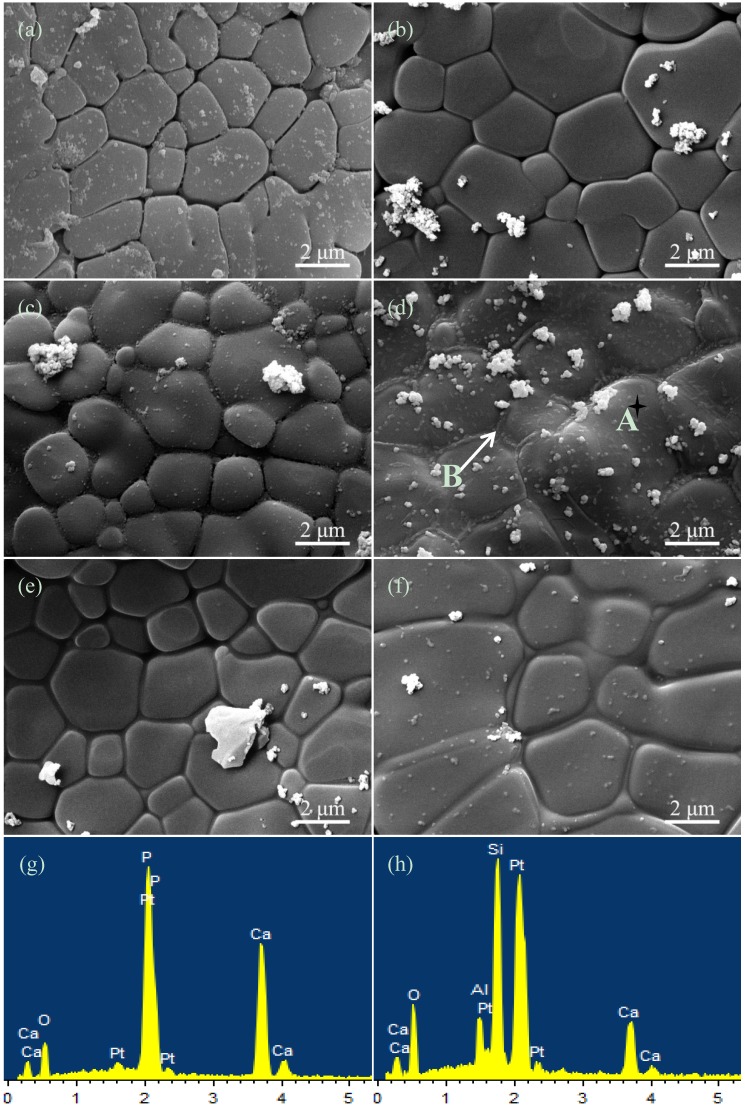
The morphologies of the HAp ceramics without or with CAS: (**a**) 0; (**b**) 1; (**c**) 2; (**d**) 3; (**e**) 4; and (**f**) 5 wt% and energy dispersive spectroscopy (EDS) spectrums: (**g**) A; (**h**) B.

The micrographs of the HAp ceramics without or with CAS were shown in Figure 2a–f, corresponding to CAS contents of 0, 1, 2, 3, 4 and 5 wt%, respectively. The grains of HAp ceramics without CAS were loose (Figure 2a), which indicated that they were not well sintered. The intergranular spacing of HAp ceramics decreased with increasing CAS from 1 to 2 wt% (Figure 2b,c). When CAS content was 3 wt%, a fully dense structure was obtained, and grain boundary phase appeared (Figure 2d). EDS analysis further demonstrated that grains were consist of Ca, P and O elements (Figure 2g) and grain boundaries were consist of Ca, Si, Al and O elements (Figure 2h), indicating CAS formed a liquid phase around the HAp grains. Moreover, the grains uniformity of the HAp ceramics with 3 wt% CAS significantly improved compared with that of other compositions. The excessive grain boundary phase formed when CAS content was 4 or 5 wt% (Figure 2e,f). It was evident to illustrate that CAS liquid phase could wet HAp crystalline grain and promote densification. The grains shape transformed from polygon to round shape gradually, which indicated the HAp grains dissolved and transformed in sintering process.

It was obvious that CAS could form a viscous flow under the sintering conditions. In liquid phase sintering theory [23], the solid particles were completely wetted by the viscous flow, which generated abundant capillary force between the particles. It facilitated densification via rearranging the particles to produce better arrangement, and via providing a rapid spread channel between the particles to realize rapid mass transfer in dissolution-reprecipitation process. According to the micrographs, though there was no evidence of reprecipitation, CAS formed a viscous flow during sintering.

### 2.2. Mechanical Characterization

**Figure 3:**
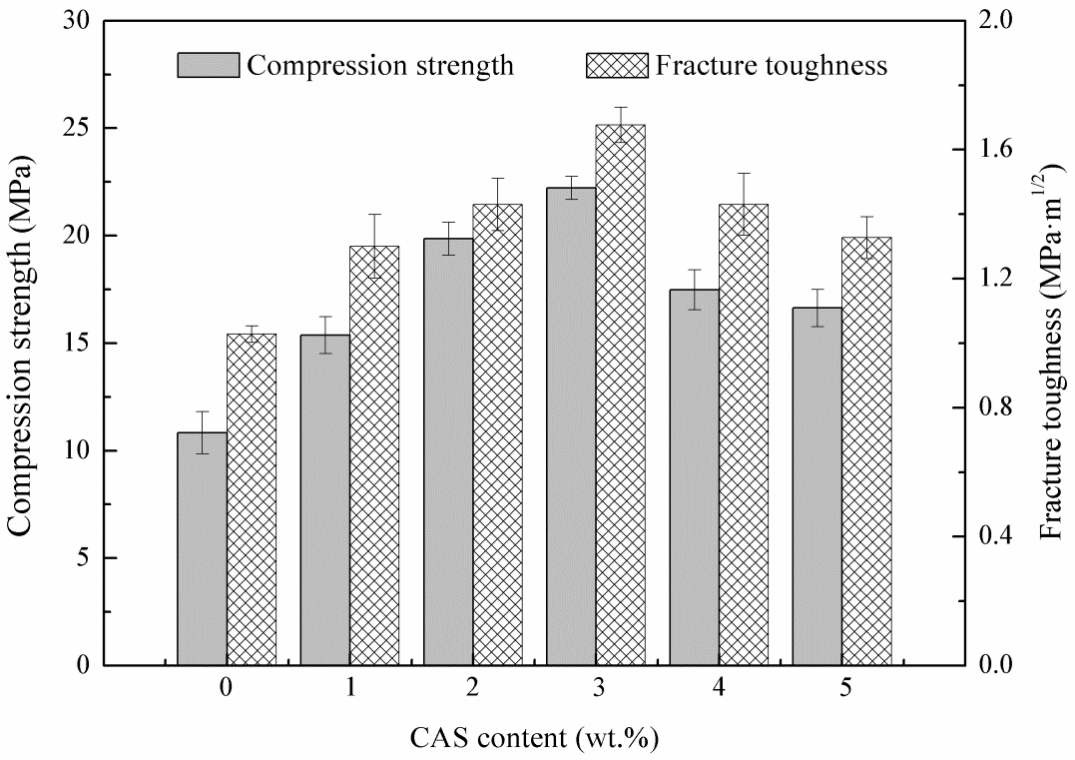
Compression strength and fracture toughness of the HAp ceramics as a function of CAS.

The effects of CAS content on the compression strength and fracture toughness of the HAp ceramics were illustrated in Figure 3. According to the histogram, both the compression strength and fracture toughness plots showed a similar trend. The compression strength and fracture toughness were enhanced when CAS was added. The optimal compression strength and fracture toughness were achieved for the HAp ceramics with 3 wt% CAS, reaching 22.22 ± 0.77 MPa and 1.68 ± 0.06 MPa·m^1/2^, respectively, which represented 105% improvement in compression strength and 63% improvement in fracture toughness compared with those of the HAp ceramics without CAS. However, both of the compression strength and fracture toughness were decreased with further increasing CAS.

The results indicated that CAS had a positive effect on the compression strength and fracture toughness. The improvement of compression strength and fracture toughness was attributed to uniform grains and improved densification, which was achieved through the solid particle rearrangement caused by capillary force during liquid phase sintering [32]. The more additive doped, the better densification was. Moreover, if the ceramics were sintered to nearly full density, the presence of CAS decreased the mechanical properties due to the excessive grain boundary phase, which separated HAp grains [33]. The HAp ceramics with 3 wt% CAS exhibited favorable mechanical properties; so, they were chosen to perform *in vitro* bioactivity and biocompatibility tests.

### 2.3. SBF Study

**Figure 4:**
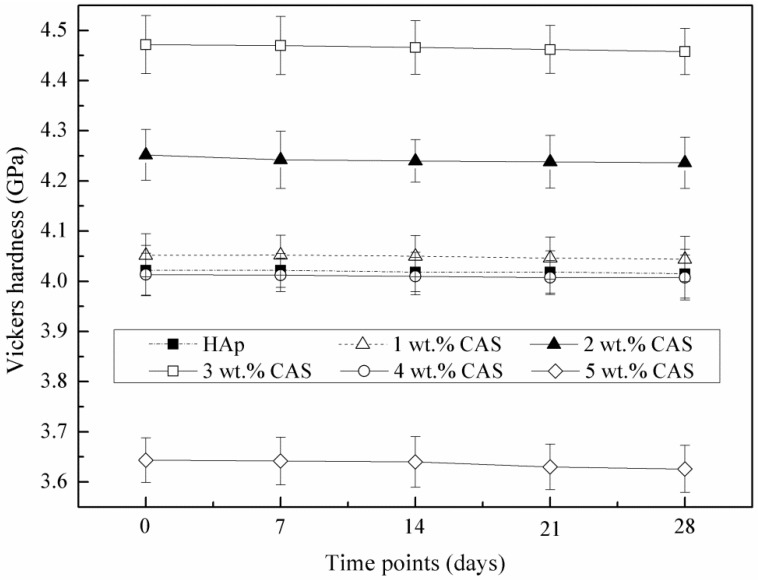
Vickers hardness of the HAp ceramics as a function of immersion time in simulated body fluid (SBF).

The average Vickers hardness of the HAp ceramics without or with CAS was shown in Figure 4. The Vickers hardness was increased with increasing CAS from 0 to 3 wt%. However, it decreased with further increasing CAS to 4 or 5 wt%. The maximum Vickers hardness of 4.47 ± 0.05 GPa (an increase of 11% over that of the HAp ceramics without CAS) was obtained with 3 wt% CAS. The results showed a similar variation with the compression strength and fracture toughness. After immersion in SBF at different time, the Vickers hardness was decreased slightly, which indicated that the HAp ceramics could maintain the mechanical properties even if they were immersed in SBF. In general, to maintain mechanical properties during immersion period, it was necessary to develop the bone scaffolds with controlled mechanical properties loss [34].

**Figure 5:**
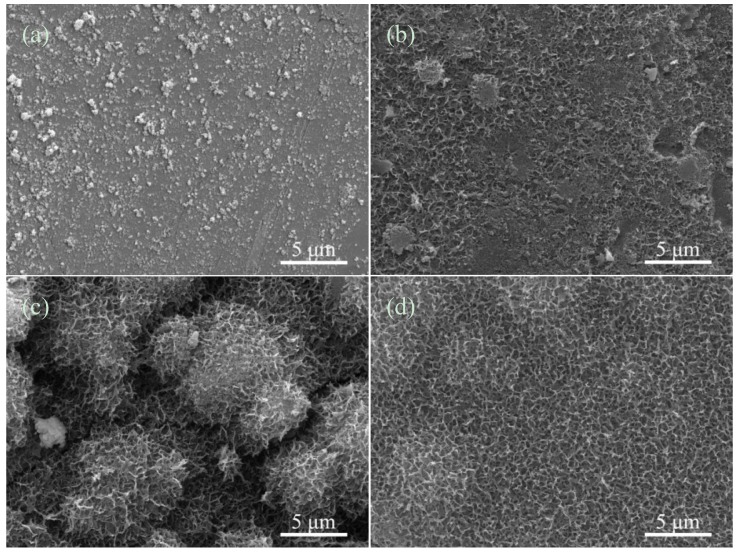
The HAp ceramics with 3 wt% CAS after immersion in SBF for (**a**) 7; (**b**) 14; (**c**) 21; and (**d**) 28 days.

The morphologies of apatite on the HAp ceramics with 3 wt% CAS after immersion in SBF at different time were observed by SEM (Figure 5). At initial stage of immersion, some precipitations formed and dispersed on the HAp ceramics surface (Figure 5a). After immersion for 14 days, the precipitation nucleated whilst apatite almost covered on the HAp ceramics surface completely (Figure 5b). At day 21 after soaking in SBF, the porous hemispherical pellets were observed (Figure 5c). After 28 days, these hemispherical pellets were further connected to each other and formed a sponge-like apatite layer (Figure 5d). The bone-like apatite layer formed as consequence of the dissolution and precipitation process of the HAp ceramics [35]. Besides, Ca, P and Si ions were released from the HAp ceramics and presented nucleus to form the apatite. The results of SBF study indicated that the HAp ceramics with 3 wt% CAS had an outstanding bioactivity.

### 2.4. Cell Culture

**Figure 6:**
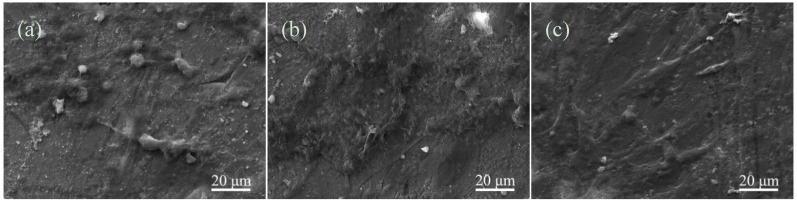
The MG-63 cells cultured on the HAp ceramics with 3 wt% CAS for (**a**) 1; (**b**) 3; and (**c**) 5 days.

Osteoblast-like MG-63 cells were cultured on the HAp ceramics for 1, 3 and 5 days, respectively. The micromorphological features of cells after cultivation were shown in Figure 6. At day 1 after seeding, the MG-63 cells adhered with pseudopodia on the HAp ceramics, and exhibited a spherical morphology (Figure 6a). After 3 days, the cells changed to flat morphology. Besides, extracellular matrix, which was secreted by the MG-63 cells, appeared and covered on the HAp ceramics (Figure 6b). With the culture time increasing to 5 days, the HAp ceramics were completely covered with the cells and extracellular matrix (Figure 6c). Simultaneously, the cells exhibited elongated and flat morphology, and proliferated well on the HAp ceramics. This *in vitro* experiment indicated that the HAp ceramics with 3 wt% CAS possessed excellent biocompatibility in terms of the MG-63 cells culturing.

## 3. Experimental Section

### 3.1. Materials and Method

HAp composite powders with different amounts of CAS were prepared in following steps. Firstly, CAS was prepared by mixing 23.3 wt% CaO (Kemiou Chemical Reagent Co., Tianjin, China), 14.7 wt% Al_2_O_3_ (Wanjing New Material Co., Hangzhou, China) and 62.0 wt% SiO_2_ (Emperor Nano Material Co., Nanjing, China) in an appropriate amount of anhydrous alcohol followed by ultrasonic dispersion for 50 min. The dispersed powders were put in the drying oven at 60 °C for 8 h. Afterwards, they were ground into fine powders by using a mortar and pestle. Commercially available medical grade HAp (Emperor Nano Material Co., Nanjing, China) was used for this research. Finally, 1, 2, 3, 4 and 5 wt% of CAS were added into HAp, respectively, followed by ball milling for 6 h.

**Figure 7:**
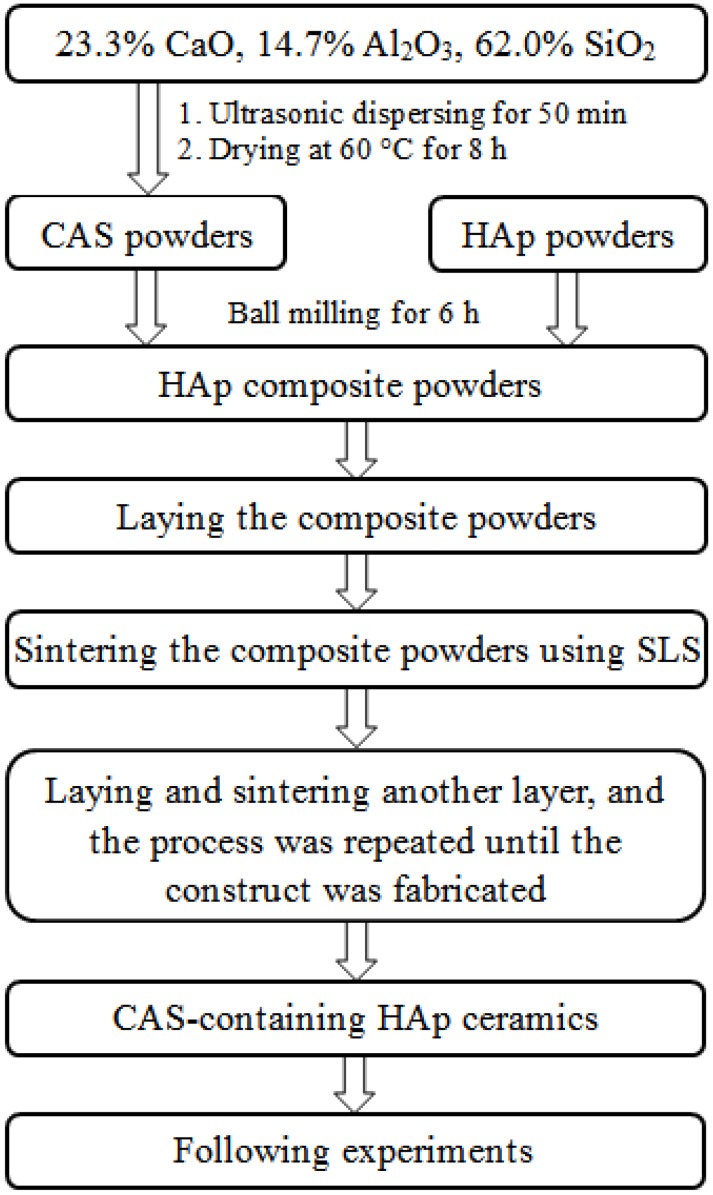
Scheme for the preparation of CAS-containing HAp.

A home-made SLS system was used in this study [36]. In the SLS system, the scaffolds preparation was conducted using the following procedure: firstly, the prepared HAp composite powders with a layer thickness of 0.1–0.2 mm were laid on working platform. Secondly, the powders were sintered selectively using a focused laser beam. The sintering track was controlled directly by computer-aided design (CAD). Thirdly, the working platform moved down with a layer’s height while the sintering of former layer was completed. Subsequently, the powders were laid and sintered repeatedly. Finally, when sintering was completed, three-dimensional scaffolds were obtained after brushing off the unsintered powders externally and internally. All sintering parameters were remained constant during sintering and shown in Table 1. The scheme of the preparation of CAS-containing HAp was presented in Figure 7.

**Table 1:** Parameters setting for selective laser sintering.

Parameters	Spot Diameter (mm)	Scan Spacing (mm)	Laser Power (W)	Scan Speed (mm/min)	Layer Thickness (mm)
Value	1.0	2.0	6	100	0.1–0.2

### 3.2. Microstructural Analysis

The HAp ceramics without or with CAS were etched using 5% hydrofluoric acid solution for 5 min. The initial powders and HAp ceramics were coated with gold for 100 s in a sputtering machine (JFC-1600 auto fine coater, JEOL Ltd., Tokyo, Japan). Scanning electron microscopy (SEM, TESCAN MIRA3 LMU, Co., Brno, Czech) was used to observe and analyze the microstructure of the initial powders and HAp ceramics. Energy dispersive spectroscopy (EDS, Oxford X-Max20, Inc., Oxford, UK) was performed for chemical microanalysis.

### 3.3. Mechanical Characterization

The compression strength, fracture toughness and Vickers hardness of the HAp ceramics with different contents of CAS were investigated. The compression strength was tested by a universal testing machine (Zhuoji Instruments Co., Ltd., Shanghai, China) with the crosshead speed of 0.5 mm/min. The compression strength was determined by the stress-strain curve obtained from the compression test.

The Vickers hardness was tested on the polished surface of the HAp ceramics using a Vickers microhardness tester (HXD-1000TM/LCD, Digital Micro Hardness Tester, Taiming Optical Instrument Co., Ltd., Shanghai, China) with a maximum load of 4.9 N and an interaction time of 15 s. In the test, the indentation was obtained with a Vickers diamond indenter. The fracture toughness was calculated by the Equation (1) [37]:

*K_IC_* =0.0824(*P*/*c*^3/2^) (1)

where *K_IC_* is the fracture toughness (MPa·m^1/2^); *P* is the indentation load (N); and *c* is the induced radial crack length (m). In this study, five specimens with each composition were performed to get an average compression strength, fracture toughness and Vickers hardness. The results were recorded as means ± standard deviation.

### 3.4. SBF Study

SBF has been used widely to study the *in vitro* bioactivity of bioceramic materials by examining the formation of bone-like apatite layer, which played an important impact on the tissue adhesion [38]. In this study, SBF solution was prepared according to the process introduced by Kokubo *et al.* [39]. The ion concentrations of SBF were similar to human body plasma as shown in Table 2. The HAp ceramics were immersed in SBF solution, which was replaced every day. The HAp ceramics were extracted from SBF after 7, 14, 21 and 28 days, respectively, followed by cleaning out with distilled water and dried at 60 °C. After immersion in SBF, the Vickers hardness of the HAp ceramics was measured; the morphologies of the HAp ceramics were observed under SEM.

**Table 2:** The ions concentration of human blood plasma and SBF.

Ions	Ions Concentration (mmol·L^−1^)							
	Na^+^	K^+^	Ca^2+^	Mg^2+^	Cl^−^	HPO_4_^2−^	HCO_3_^−^	SO_4_^2−^
Blood plasma	142.0	5.0	2.5	1.5	103.0	1.0	27.0	0.5
SBF	142.0	5.0	2.5	1.5	147.8	1.0	4.2	0.5

### 3.5. Cell Culture

**Figure 8:**
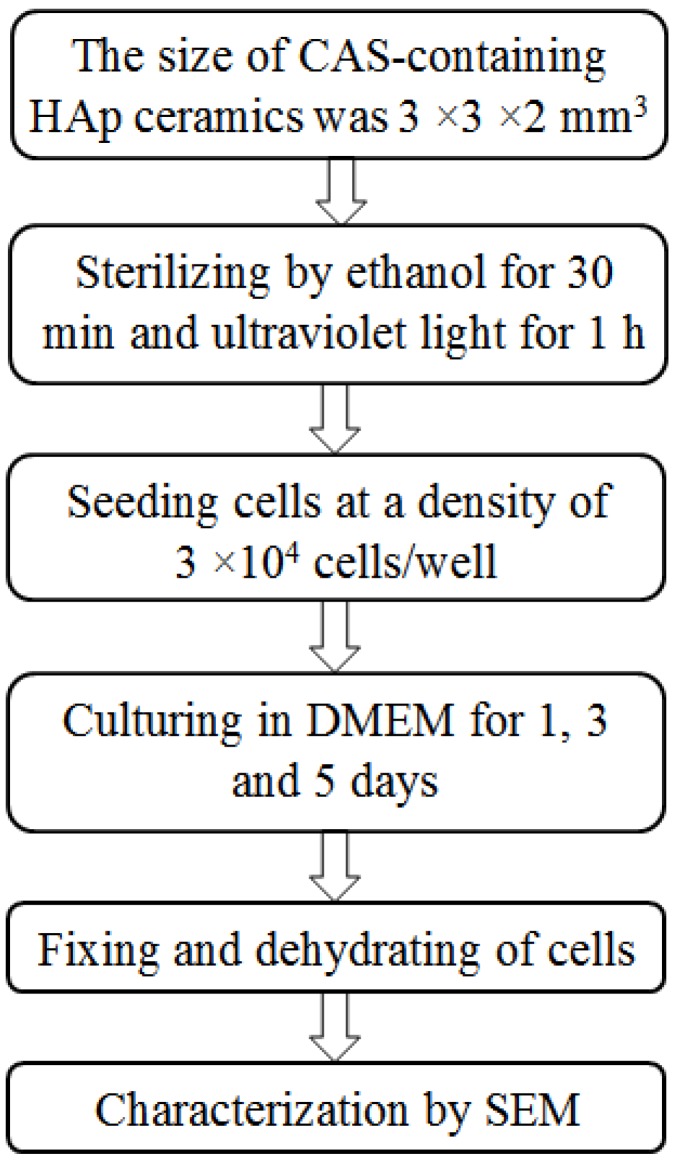
Scheme for cultivation of MG-63 cells.

Osteoblast-like MG-63 cells were used to investigate the biocompatibility of the HAp ceramics. Before seeding, the HAp ceramics were sterilized by 70% ethanol for 30 min and then further sterilized by ultraviolet light (UV) for 1 h. Cells were seeded on the HAp ceramics at a density of 3 × 10^4^ cells/well in a 12-well plate. Cultivation was performed in Dulbecco’s Modified Eagle’s Medium (DMEM) supplementing with 10% fetal bovine serum (FBS), and maintained in a humidified condition containing 5% CO_2_ at 37 °C for 1, 3 and 5 days, respectively. After every culture time, the HAp ceramics were extracted from the DMEM and washed with phosphate buffered saline (PBS) to remove non-adherent cells. The cells were fixed in 2.5% glutaraldehyde for 40 min and dehydrated in an alcohol concentration gradient (70%, 80%, 90% and 100%) for 10 min, respectively. The morphologies of MG-63 cells after different culture time were observed under SEM. The scheme of cultivation of MG-63 cells was presented in Figure 8.

## 4. Conclusions

In this paper, the HAp ceramics with different contents of CAS (0, 1, 2, 3, 4 and 5 wt%) were prepared by SLS. Mechanical properties of the HAp ceramics were improved significantly by adding a small quantity of CAS as a liquid phase. The mechanical properties with 105% increase in compression strength, 63% increase in fracture toughness and 11% increase in Vickers hardness were obtained. The improvement of mechanical properties was attributed to the improved densification. However, mechanical properties were decreased, while 4 or 5 wt% CAS was added, which was due to the excessive liquid phase. Moreover, *in vitro* bioactivity study carried out in SBF solutions showed the formation of bone-like apatite layer on the HAp ceramics. Meanwhile, the Vickers hardness of the HAp ceramics was decreased slightly after immersion in SBF, indicating excellent mechanical stability. In addition, cell culture was performed with the MG-63 cells, and the results indicated that the cells adhered and spread well. Therefore, the HAp ceramics with CAS were bioactive and biocompatible substitutes, which might be suitable for bone repair.

## References

[1] Soucacos P.N., Kokkalis Z.T., Piagkou M., Johnsonb E.O. (2013). Vascularized bone grafts for the management of skeletal defects in orthopaedic trauma and reconstructive surgery. Injury.

[2] Schlickewei W., Schlickewei C. (2007). The use of bone substitutes in the treatment of bone defects–The clinical view and history. Macromol. Symp. Aug..

[3] Shi Y., Niedzinski J.R., Samaniego A., Bogdansky S., Atkinson B.L. (2012). Adipose-derived stem cells combined with a demineralized cancellous bone substrate for bone regeneration. Tissue Eng. Part A.

[4] Goodridge R.D., Dalgarno K.W., Wood D.J. (2006). Indirect selective laser sintering of an apatite-mullite glass-ceramic for potential use in bone replacement applications. Proc. Inst. Mech. Eng. H.

[5] Kaur G., Pandey O.P., Singh K., Homa D., Scott B., Pickrell G. (2014). A review of bioactive glasses: Their structure, properties, fabrication and apatite formation. J. Biomed. Mater. Res. A.

[6] Martínez-Vázquez F.J., Pajares A., Guiberteau F., Miranda P. (2014). Effect of polymer infiltration on the flexural behavior of β-tricalcium phosphate robocast scaffolds. Materials.

[7] Lawrence B.J., Madihally S.V. (2008). Cell colonization in degradable 3D porous matrices. Cell Adhes. Migr..

[8] Guan S., Zhang X.L., Lin X.M., Liu T.Q., Ma X.H., Cui Z.F. (2013). Chitosan/gelatin porous scaffolds containing hyaluronic acid and heparan sulfate for neural tissue engineering. J. Biomater. Sci.-Polym. Ed..

[9] Murphy C.M., Haugh M.G., O’Brien F.J. (2010). The effect of mean pore size on cell attachment, proliferation and migration in collagen–glycosaminoglycan scaffolds for bone tissue engineering. Biomaterials.

[10] Zhou H., Lee J. (2011). Nanoscale hydroxyapatite particles for bone tissue engineering. Acta Biomater..

[11] Sun F., Koh K., Ryu S.C., Han D.W., Lee J. (2012). Biocompatibility of nanoscale hydroxyapatite-embedded chitosan films. Bull. Korean. Chem. Soc..

[12] Yang Y.H., Liu C.H., Liang Y.H., Lin F.H., Wu K.C.W. (2013). Hollow mesoporous hydroxyapatite nanoparticles (hmHANPs) with enhanced drug loading and pH-responsive release properties for intracellular drug delivery. J. Mater. Chem. B.

[13] Wu K.C.W., Yang Y.H., Liang Y.H., Chen H.Y., Sung E., Yamauchi Y., Lin F.H. (2011). Facile synthesis of hollow mesoporous hydroxyapatite nanoparticles for intracellular bio-imaging. Curr. Nanosci..

[14] Li Z., Wen T., Su Y., Wei X., He C., Wang D. (2014). Hollow hydroxyapatite spheres fabrication with three-dimensional hydrogel template. Cryst. Eng. Comm..

[15] Bastakoti B.P., Hsu Y.C., Liao S.H., Wu K.C.W., Inoue M., Yusa S.I., Yamauchi Y. (2013). Inorganic–organic hybrid nanoparticles with biocompatible calcium phosphate thin shells for fluorescence enhancement. Chem. Asian J..

[16] Chevalier J., Gremillard L. (2009). Ceramics for medical applications: A picture for the next 20 years. J. Eur. Ceram. Soc..

[17] Lin K., Chen L., Chang J. (2012). Fabrication of dense hydroxyapatite nanobioceramics with enhanced mechanical properties via two-step sintering process. Int. J. Appl. Ceram. Technol..

[18] Reves B.T., Jennings J.A., Bumgardner J.D., Haggard W.O. (2011). Osteoinductivity assessment of BMP-2 loaded composite chitosan-nano-hydroxyapatite scaffolds in a rat muscle pouch. Materials.

[19] Tripathi G., Basu B. (2012). A porous hydroxyapatite scaffold for bone tissue engineering: Physico-mechanical and biological evaluations. Ceram. Int..

[20] Duan B., Wang M. (2011). Selective laser sintering and its application in biomedical engineering. MRS Bull..

[21] Sandler N., Lammens R.F. (2011). Pneumatic dry granulation: Potential to improve roller compaction technology in drug manufacture. Expert Opin. Drug Del..

[22] Bouslama N., Chevalier Y., Bouaziz J., Ayed F.B. (2013). Influence of the sintering temperature on Young’s modulus and the shear modulus of tricalcium phosphate–fluorapatite composites evaluated by ultrasound techniques. Mater. Chem. Phys..

[23] Lupulescu A., Glicksman M.E. (2000). Diffusion-limited crystal growth in silicate systems: Similarity with high-pressure liquid-phase sintering. J. Cryst. Growth.

[24] Wei W., Chen K., Ge G. (2013). Strongly coupled nanorod vertical arrays for plasmonic sensing. Adv. Mater..

[25] Hotta M., Hojo J. (2010). Inhibition of grain growth in liquid-phase sintered SiC ceramics by AlN additive and spark plasma sintering. J. Eur. Ceram. Soc..

[26] Liu D., Zhuang J., Shuai C., Peng S. (2013). Mechanical properties’ improvement of a tricalcium phosphate scaffold with poly-L-lactic acid in selective laser sintering. Biofabrication.

[27] Batra U., Kapoor S., Sharma J.D. (2011). Nano-Hydroxyapatite/Fluoridated and Unfluoridated Bioactive Glass Composites: Structural Analysis and Bioactivity Evaluation. Proceedings of the International Conference on Advances in Condensed and Nano Materials (ICACNM-2011).

[28] Oktar F.N., Agathopoulos S., Ozyegin L.S., Gunduz O., Demirkol N., Bozkurt Y., Salman S. (2007). Mechanical properties of bovine hydroxyapatite (BHA) composites doped with SiO_2_, MgO, Al_2_O_3_, and ZrO_2_. J. Mater. Sci.: Mater. Med..

[29] Bellucci D., Cannillo V., Sola A. (2011). A new highly bioactive composite for scaffold applications: A feasibility study. Materials.

[30] Nath S., Biswas K., Wang K., Bordia R.K., Basu B. (2010). Sintering, phase stability, and properties of calcium phosphate-mullite composites. J. Am. Ceram. Soc..

[31] Bose S., Tarafder S., Banerjee S.S., Davies N.M., Bandyopadhyay A. (2011). Understanding *in vivo* response and mechanical property variation in MgO, SrO and SiO_2_ doped β-TCP. Bone.

[32] German R.M., Suri P., Park S.J. (2009). Review: Liquid phase sintering. J. Mater. Sci..

[33] Borrero-López O., Ortiz A.L., Guiberteau F., Padture N.P. (2007). Effect of liquid-phase content on the contact-mechanical properties of liquid-phase-sintered α-SiC. J. Eur. Ceram. Soc..

[34] Bhatt H.A., Kalita S.J. (2007). Influence of oxide-based sintering additives on densification and mechanical behavior of tricalcium phosphate (TCP). J. Mater. Sci.: Mater. Med..

[35] Ribeiro C., Rigo E.C.S., Sepúlveda P., Bressiani J.C., Bressiani A.H.A. (2004). Formation of calcium phosphate layer on ceramics with different reactivities. Mater. Sci. Eng. C.

[36] Shuai C.J., Feng P., Gao C.D., Zhou Y., Peng S.P. (2011). Simulation of temperature field during the laser sintering process of nano-hydroxyapatite powder. Adv. Mater. Res..

[37] Veljovic D., Palcevskis E., Zalite I., Petrovic R., Janackovic D. (2013). Two-step microwave sintering-A promising technique for the processing of nanostructured bioceramics. Mater. Lett..

[38] Kaur G., Pickrell G., Kimsawatde G., Homa D., Allbee H.A., Sriranganathan N. (2014). Synthesis, cytotoxicity, and hydroxyapatite formation in 27-Tris-SBF for sol-gel based CaO-P_2_O_5_-SiO_2_-B_2_O_3_-ZnO bioactive glasses. Sci. Rep..

[39] Kokubo T., Kushitani H., Sakka S., Kitsugi T., Yamamuro T. (1990). Solutions able to reproduce *in vivo* surface-structure changes in bioactive glass-ceramic A-W^3^. J. Biomed. Mater. Res..

